# A Semiquantitative Scoring System for Histopathological and Immunohistochemical Assessment of Lesions and Tissue Tropism in Avian Influenza

**DOI:** 10.3390/v13050868

**Published:** 2021-05-09

**Authors:** Maria Landmann, David Scheibner, Annika Graaf, Marcel Gischke, Susanne Koethe, Olanrewaju I. Fatola, Barbara Raddatz, Thomas C. Mettenleiter, Martin Beer, Christian Grund, Timm Harder, Elsayed M. Abdelwhab, Reiner Ulrich

**Affiliations:** 1 Institute of Veterinary Pathology, Leipzig University, 04103 Leipzig, Germany; Maria.Landmann@vetmed.uni-leipzig.de (M.L.); barbara.raddatz@abbvie.com (B.R.); 2 Institute of Molecular Virology and Cell Biology, Friedrich-Loeffler-Institut, Federal Research Institute for Animal Health, 17493 Greifswald-Insel Riems, Germany; David.Scheibner@fli.de (D.S.); 1000000075.gast@fli.de (M.G.); ThomasC.Mettenleiter@fli.de (T.C.M.); El-SayedMohammed.AbdEl-Whab@fli.de (E.M.A.); 3 Institute of Diagnostic Virology, Friedrich-Loeffler-Institut, Federal Research Institute for Animal Health, 17493 Greifswald-Insel Riems, Germany; Annika.Graaf@fli.de (A.G.); Susanne.Koethe@fli.de (S.K.); Martin.Beer@fli.de (M.B.); Christian.Grund@fli.de (C.G.); Timm.Harder@fli.de (T.H.); 4 Institute for Novel and Emerging Infectious Diseases, Friedrich-Loeffler-Institut, Federal Research Institute for Animal Health, 17493 Greifswald-Insel Riems, Germany; fatolan@yahoo.com

**Keywords:** avian influenza virus, poultry, histopathology, immunohistochemistry, semiquantitative scoring system

## Abstract

The main findings of the post-mortem examination of poultry infected with highly pathogenic avian influenza viruses (HPAIV) include necrotizing inflammation and viral antigen in multiple organs. The lesion profile displays marked variability, depending on viral subtype, strain, and host species. Therefore, in this study, a semiquantitative scoring system was developed to compare histopathological findings across a wide range of study conditions. Briefly, the severity of necrotizing lesions in brain, heart, lung, liver, kidney, pancreas, and/or lymphocytic depletion in the spleen is scored on an ordinal four-step scale (0 = unchanged, 1 = mild, 2 = moderate, 3 = severe), and the distribution of the viral antigen in parenchymal and endothelial cells is evaluated on a four-step scale (0 = none, 1 = focal, 2 = multifocal, 3 = diffuse). These scores are used for a meta-analysis of experimental infections with H7N7 and H5N8 (clade 2.3.4.4b) HPAIV in chickens, turkeys, and ducks. The meta-analysis highlights the rather unique endotheliotropism of these HPAIV in chickens and a more severe necrotizing encephalitis in H7N7-HPAIV-infected turkeys. In conclusion, the proposed scoring system can be used to condensate HPAIV-typical pathohistological findings into semiquantitative data, thus enabling systematic phenotyping of virus strains and their tissue tropism.

## 1. Introduction

Influenza A virus (IAV), a genus within the *Orthomyxoviridae* family, is further classified based on the antigenicity of the surface proteins hemagglutinin (HA) and neuraminidase (NA) into different HxNy subtypes [1].

At present, 18 HA (H1–H18) and 11 NA (N1–N11) subtypes are differentiated [2]. Except for two bat-derived influenza viruses (H17N10 and H18N11), all subtypes were initially isolated from birds and are classified as avian influenza viruses (AIV) [3]. AIV shows a high variability resulting from molecular changes mainly by two mechanisms: The accumulation of point mutations (antigenic drift, especially if HA is affected [4]) and reassortment of viral gene segments during co-infection with different AIV (antigenic shift, if HA and/or NA are affected) [3,5]. However, even identical AIV subtypes exhibit highly variable pathogenicity and virulence even in closely related avian species [6,7].

According to their pathogenicity in chickens, AIV is classified into low-pathogenic (LP) and high-pathogenic (HP) strains [3]. While all H1–H16 AIV subtypes are LP, some low pathogenic avian influenza viruses (LPAIV) of H5 and H7 subtypes may shift to highly pathogenic avian influenza viruses (HPAIV). The LPAIV strains possess a HA monobasic cleavage site, which is recognized and activated by tissue-restricted trypsin-like enzymes in the aerodigestive tract. Therefore, LPAIV causes mild or no clinical signs. Conversely, the HA of HPAIV comprises polybasic cleavage site motifs, which are recognized and activated by ubiquitous furin-like enzymes. HPAIV replicates systemically, causing multiorgan dysfunction and high mortality [8,9].

Examining the pathologic findings associated with AIV infections across different hosts is vital for understanding the virulence of a particular subtype for a specific species. In general, the results of gross and histopathologic examinations are reported as morphologic diagnoses. Usually, the following components are used to characterize a lesion: type of process, the character in case of inflammation, distribution, age, severity, and special features.

The main type and character of the pathogenetic process in HPAIV-infections is virus-induced necrosis, synonymously termed necrotizing inflammation, affecting multiple organs. However, necrotizing inflammation can be highly variable, depending on factors such as viral subtype, strain, and host species. Most prominent lesions, viral antigen and distinct strain- and species-dependent differences are found in brain, lung, heart, pancreas, adrenal gland, liver, kidney, upper respiratory tract, and lymphoid organs (for H5-infected chickens, see, e.g., [10,11,12,13,14], H7-infected chickens, e.g., [15,16,17], H5-infected turkeys, e.g., [11], H5-infected ducks, e.g., [12,18,19,20,21,22], H5-infected wild birds and other waterfowl, e.g., [19,20,21,23,24,25,26]). Furthermore, HPAIV antigen is often present in endothelial cells to a varying extent, mainly, but not exclusively, in galliform species [16,17,25,27].

The distribution represents the spatial pattern of lesions and is commonly divided into the following basic categories: focal (one single lesion), multifocal (more than one lesion), and diffuse (lesion affecting nearly the whole tissue). Those terms can be varied slightly for greater accuracy; for example, the observation of only a few, sporadic foci can be termed “oligofocal,” numerous, evenly distributed foci can be termed “disseminated,” and large, converging foci can be termed “coalescing.”

Severity reflects the extent of a lesion, and often multiple criteria are taken into account, e.g., the portion of the organ affected, distribution, or complexity, and further elements of changes [28,29]. Commonly, severity grades are termed “mild,” “moderate,” and “severe,” and sometimes intermediate degrees, such as “minimal” are added.

Different approaches for histopathological and immunohistochemical examination and evaluation in AIV-infected animals have been made: Descriptive reporting of morphologic diagnoses and antigen distribution without further scoring or statistical analysis is very common [12,19,24,30], as well as basic scoring systems simply reflecting the subjective histopathologic grade of severity or amount of viral antigen without further specification of criteria and with or without additional descriptive classification [11,16,31,32,33], thereby assessing a various range of organs and lesions. Some studies also use alternative methods, e.g., counting immunoreactive cells per tissue section [34] or combining multiple scores for intensity and area of inflammation and necrosis in few specific organs [35].

Scoring of histopathologic alterations on an ordinal scale is a tool to include light-microscopically obtained information in biomedical studies and to compare lesions using statistical methods [36,37]. Ordinal scales used in scoring systems produce qualitative data (also named categorical data). Even though a number may be assigned to, e.g., a certain magnitude of a lesion, the data do not represent an actual measure, as do quantitative data, but rather approximate or characterize the alterations. For this reason, they are often considered “semiquantitative” in nature [29,36].

For subsequent statistical analysis, it is important to keep in mind that ordinal data do not meet the assumption of Gaussian distribution; thus, the median instead of the mean value is the most appropriate measure for central tendency, and nonparametric tests (e.g., Mann–Whitney U test, Kruskal–Wallis test) are the analysis method of choice [36,37]. In general, those tests are based on the comparison of ranks and gain more power with a larger sample size. Nonparametric tests tend to require a larger minimal sample size as compared to parametric tests to be mathematically able to reject the null hypothesis. For example, a comparison of two independent groups using a Mann–Whitney U test needs at least four samples per group to reveal a significant difference employing a *p*-value ≤ 0.05 [38], even with very large biological differences.

With this study, two aims were pursued: first, to define robust criteria for the semiquantitative scoring of lesion severity and virus antigen distribution using data and tissue samples of multiple AIV infection studies, and second, to compare results of semiquantitative scoring between those infection studies to gain new insights into pathogenesis and tissue tropism of different AIV. A flowchart with the process and key aspects of this study is given in Scheme S1.

## 2. Materials and Methods

### 2.1. AIV Infection Experiments

For the development of a semiquantitative scoring system and comparative meta-analysis, tissue samples and data available from multiple AIV infection experiments conducted at the Friedrich-Loeffler-Institut (FLI; Greifswald-Insel Riems, Germany) were used (experiments 1–20, details in Table S1). Experiments were conducted with a wide range of wild-type and modified LP and HP viruses, including different H4, H5, H7, and H9 subtypes. Part of the results and further details of those experiments have been published [39,40,41,42,43,44,45,46].

All original animal experiments were legally approved by the State Office of Agriculture, Food Safety and Fishery in Mecklenburg-Western Pomerania, Germany (LALLF M-V, registration numbers see Table S1).

### 2.2. Histopathological and Immunohistochemical Examination

Necropsies for all experiments were performed according to standard procedures under biosafety level-3 (BSL-3) conditions. Varying between individual experiments, specimens of brain, skin, nasal cavity, oral cavity, trachea, lung, air sacs, heart, thymus, glandular stomach, gizzard, duodenum, jejunum, caecum, liver, kidney, spleen, pancreas, adrenal gland, bursa fabricii, ovary/testis, and chorioallantoic membrane were taken. Samples were fixed in 4% neutral-buffered formaldehyde for ≥7 days, trimmed, processed, and embedded in paraffin wax. In total, 2–5 µm microtome slices were mounted on glass slides and stained with hematoxylin and eosin.

Immunohistochemical examination was performed on formaldehyde-fixed and paraffin-embedded (FFPE) tissue samples with the avidin-biotin-peroxidase complex method (Vector Laboratories, Burlingame, CA, USA) using a polyclonal rabbit anti-influenza A-nucleoprotein (NP) antibody [33,47,48] or monoclonal murine anti-influenza A-matrixprotein (MP) antibody [41] with 3-amino-9-ethyl-carbazol as chromogen and hematoxylin counterstain in individual experiments (see Table S1). Validated positive and negative archival tissues were used as controls, and the specific antibody was replaced by, e.g., Tris-Buffered Saline (TBS) [41] or rabbit serum [42].

### 2.3. Development of a Semiquantitative Scoring System

In all infection experiments, a basic histopathological and/or immunohistochemical scoring for lesions and distribution of viral antigen has been conducted as follows:

Parenchymal necrosis and/or necrotizing inflammation was evaluated on a scale from 0 to 3 based on the lesion severity grade (mild, moderate, severe). Score 0 is assigned to animals where no changes were detected, and score 3 classifies animals with severe lesions. In a similar manner, necrosis and apoptosis of lymphoid and reticuloendothelial cells and/or lymphoid depletion were assessed in lymphatic organs on a scale from 0 to 3.

Parenchymal immunoreactivity for IAV antigen was evaluated on a scale from 0 to 3. Score 0 is assigned to organs with no immunoreactive cells. Score 1, 2, and 3 are assigned to organs with single immunoreactive cells or single immunoreactive foci (i.e., focal to oligofocal), scattered foci (i.e., multifocal), and numerous to coalescing foci of immunoreactive cells, respectively.

Endothelial cells of the respective organs were evaluated in an analogous manner. Score 0 was assigned to animals with no immunoreactive endothelial cells. Score 1 was appointed if immunoreactive endothelial cells were detected in single blood vessels, score 2 classified organs with immunoreactive endothelial cells in multiple blood vessels, and score 3 was assigned to animals with a diffuse immunoreactivity of the endothelial cells in the respective organ.

The scoring of all samples was done by one board-certified veterinary pathologist (RU) in the previous studies. In the current study, slides from these infection experiments were used to determine the common range of lesion severity and antigen distribution across H4, H5, H7, and H9 influenza viruses in the major organs of chickens, ducks, and turkeys. Experiments were critically re-assessed concerning the universal applicability of the basic scoring system and for the definition of more detailed, quantitative scoring criteria (Table S1). Light microscopic pictures of typical lesions were taken using an Olympus BX-53 microscope equipped with 2×, 4×, 10×, 20× and 40× objectives and a DP26 digital camera (Olympus Deutschland GmbH, Hamburg, Germany).

### 2.4. Comparative Meta-Analysis between Selected Studies

A comparative meta-analysis was conducted with the basic scores for lesion severity and virus antigen distribution, which were gained as described above. Inclusion criteria for selective meta-analysis between infection experiments were chosen as follows: (1.) animals infected with HP wild-type virus or a clone of HP wild-type virus, (2.) tissues available from at least three animals per virus subtype, (3.) inoculation with defined virus doses, (4.) infection with a single virus (monoinfection), (5.) infection in hatched chickens, turkeys, or ducks, (6.) part of results published up to and including 2019. Some of the tissues available from the following experiments fulfilled inclusion criteria and were used for comparative analysis and development of a semiquantitative scoring system:

Experiment 3 [41]: Experiment 3 comprises infection with isogenic H7N7 HP virus (A/chicken/Germany/AR1385/2015) [49] in 6-week-old white leghorn chickens purchased from Lohmann Animal Health (Cuxhaven, Germany) inoculated via oculo-oronasal route with 10^1^, 10^3^, 10^4^, 10^5^, 10^5.7^, and 10^6^ mean embryo infectious doses 50 (EID_50_) HP per animal or co-housed as sentinels at 1-day post-inoculation (dpi). Histopathologic examination was performed on animals necropsied at 2 dpi. A group with all necropsied animals infected with H7N7 HP (inoculated and sentinel) is further referred to as “chicken HP H7N7 2 dpi all virus doses, inoculated and sentinel animals.” This merged group was used only for refinement of the scoring system, whereas another combined group consisting only of animals infected oculo-oronasally with high virus doses (10^5^, 10^5.7^, 10^6^ EID_50_) was used for comparative analysis because those infection dose groups showed similar behavior in clinical and virological analyses (further referred to as “chicken HP H7N7 2 dpi (high virus doses only)”).

Experiments 8 to 11 [44]: Chickens, ducks, and turkeys were inoculated with 10^5^ plaque-forming units (PFU) per animal via the oculonasal route. Infection was among others performed with a cloned H7N7 HP (A/chicken/Germany/AR1385/2015) [49] virus on 6-week-old white leghorn chickens purchased from VALO BioMedia (Osterholz-Scharmbeck, Germany), 6-week-old, commercially available white-breasted turkeys and 2- to 3-week-old Muscovy ducks from a local supplier. Here, the selected groups of HP-infected animals were necropsied at 3–4 dpi (the other groups at 2–4 dpi), and a histopathological examination was conducted. Those selected groups for comparison are further referred to as “chicken HP H7N7 3–4 dpi,” “turkey HP H7N7 4 dpi,” and “duck HP H7N7 4 dpi.”

Some of the experiments fulfilled all inclusion criteria except having enough initially infected animals per group available for histopathologic examination. To be able to compare different viral subtypes between different species, Experiment 6 was chosen for exemplary comparison, including sentinel animals.

Experiment 6 [42] included H5N8 HP clade 2.3.4.4. group B (A/tufted_duck/Germany/AR8444-L01987/2016; DE16-H5N8B) infection in adult 6- to 7-month-old “Rouen Claire” ducks obtained from a local breeder inoculated oculonasally with 10^6^ tissue culture infective doses 50 (TCID_50_). White leghorn chickens (VALO BioMedia, Osterholz-Scharmbeck, Germany) and ducks were housed together with the inoculated birds at 1 dpi and served as sentinels. Necropsy and histopathologic examination were performed for the animals that died between 3–7 days post-contact (dpc) for chickens (sentinel animals) and 4–8 dpi for ducks (two inoculated and two sentinel animals). Dpi for all ducks are counted as days since the original inoculation of non-sentinel ducks. Dpc for the two sentinel ducks can be calculated as 1 day less than dpi (e.g., 4 dpc at 5 dpi). Selected groups for comparison are further referred to as “chicken HP H5N8B 3–7 dpc,” “chicken HP H5N8B 3–4 dpc,” and “duck HP H5N8B 4–8 dpi.”

### 2.5. Quantitative Real-Time RT-PCR

For Experiment 6, molecular analysis of selected tissues was conducted for some animals, which were also subjected to histopathological and immunohistochemical examination. Analysis was performed via reverse transcription-quantitative polymerase chain reaction (RT-qPCR) specific for the M gene [42,50].

### 2.6. Statistical Analysis

Ordinal data were analyzed using Mann–Whitney U tests for two group comparisons or Kruskal–Wallis tests for the comparison of three groups, followed by Dunn’s post-hoc-tests for subsequent pairwise comparisons (GraphPad, version 8.2.0 for Windows, GraphPad Software, San Diego, CA, USA). Statistical significance was generally accepted as *p* ≤ 0.05. For comparison of RT-qPCR results of Experiment 6 [42] with immunohistochemical parenchyma virus scores of selected tissues, Spearman’s correlation analysis was conducted (GraphPad, version 8.2.0 for Windows, GraphPad Software, San Diego, CA, USA).

## 3. Results

As the first aim of this study, we propose an elaborated scoring system with a detailed explanation of scoring criteria, which was developed by critical reassessment of data and tissues from multiple infection experiments with subtype H4, H5, H7, and H9 viruses in chickens, ducks, turkeys, geese, and mammals (Table S1).

Furthermore, as a second aim, we perform a meta-analysis comparing semiquantitative scores—gained as described above—across studies, viruses, and host species to win new insights into pathogenesis and tissue tropism of different AIV.

Results of the histopathological and immunohistochemical examination and the basic semiquantitative scoring have been published in multiple independent manuscripts for most of the infection experiments previously (see [39,40,41,42,43,44,45,46]).

### 3.1. Lesion Score: Histopathologic Scoring for the Grade of AIV-Induced Lesions

Basic histopathologic scores of HPAIV-infected animals from Experiments 3, 6 and 8 to 11 were most pronounced and frequently found in brain, heart, liver, respiratory tract, pancreas, adrenal gland, and kidney and detected sporadically in the glandular stomach, duodenum, and gonads of single animals. Lymphatic depletion or necrosis was present throughout all examined lymphoid organs (Figure S1a,b).

**Figure 1:**
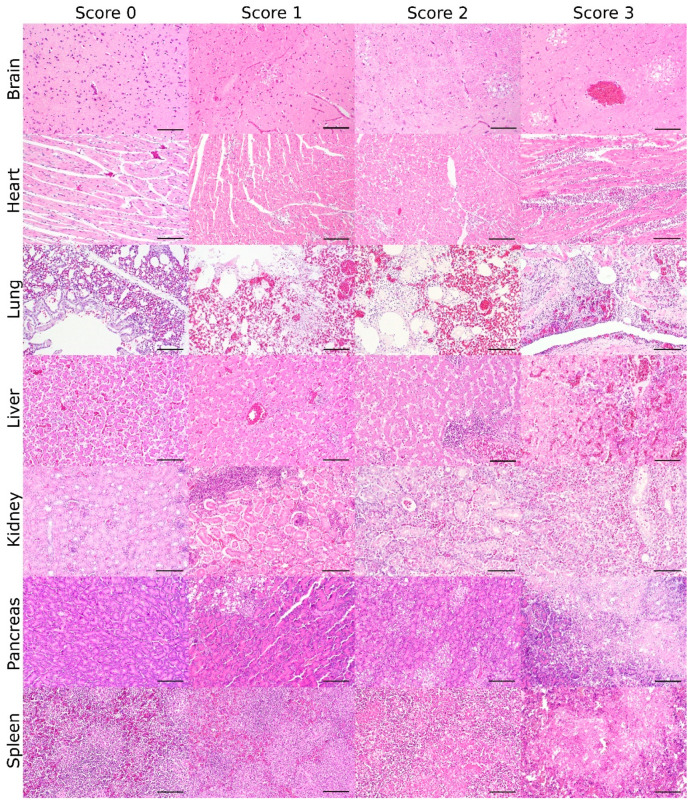
Lesion score: Histopathologic scoring of the grade of avian influenza virus (AIV)-induced necrotizing lesions and lymphoid depletion in AIV-infected birds (bar = 100 µm, hematoxylin and eosin).

The proposed scoring system focuses on the assessment of the brain, heart, liver, kidney, lung, pancreas, and spleen, as they represent the great parenchymal organs and lymphoid tissues mainly affected. Scoring criteria are defined and illustrated for those organs in Table 1 and Figure 1.

### 3.2. Virus Score: Immunohistochemical Scoring for the Distribution of AIV Antigen

**Figure 2:**
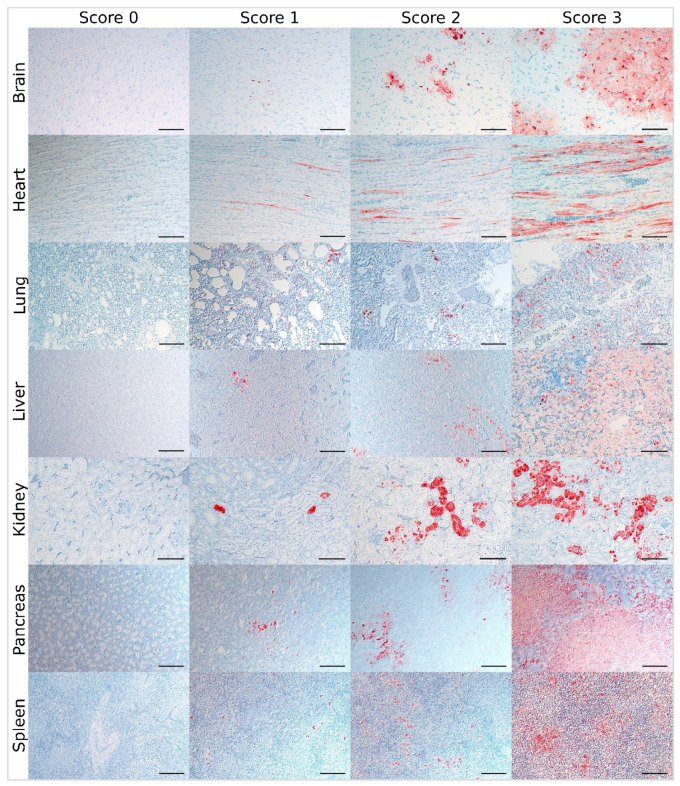
Virus score: Immunohistological scoring of influenza A virus (IAV)-antigen distribution, avian influenza virus (AIV)-infected birds (bar = 100 µm, IAV-matrixprotein immunohistochemistry, avidin-biotin-peroxidase complex method, 3-amino-9-ethyl-carbazol as chromogen and hematoxylin counterstain; Nomarski contrast).

In HPAIV-infected animals from Experiments 3, 6, and 8 to 11, parenchymal virus antigen was present in most of the examined tissues and frequently associated with histopathological lesions (Figure S1c,d). Therefore, the refined immunohistochemical scoring system proposed here focuses on the assessment of the brain, heart, liver, kidney, lung, pancreas, and spleen in accordance with the lesion score. The cells to be considered as parenchymal cells for those organs are listed in Table 2. Furthermore, for those tissues, improved scoring criteria for the classification of inconclusive cases were subsequently extracted and illustrated by re-evaluation of representative data and slides as well (Table 3, Figure 2).

Immunoreactive cells other than parenchymal cells should be excluded from the rating, but immunoreactive cellular debris included if located among parenchymal cells and associated with necrotic lesions. For NP, only cells with a clear intranuclear immunohistochemical staining pattern should be scored, while for MP, cells with cytoplasmic staining pattern and typical pathomorphology (e.g., macrophages) and cells with an intranuclear signal should be taken into account. Inconclusive cells should be excluded from the evaluation.

Basic scoring of endothelial cells of the respective organs was valued as sufficient and in no need of further explanation (immunoreactive endothelial cells—0: none, 1: in single blood vessels, 2: in multiple blood vessels, 3: diffuse immunoreactivity).

### 3.3. Comparison of H7N7-Infected Chickens, Turkeys, and Ducks

**Figure 3:**
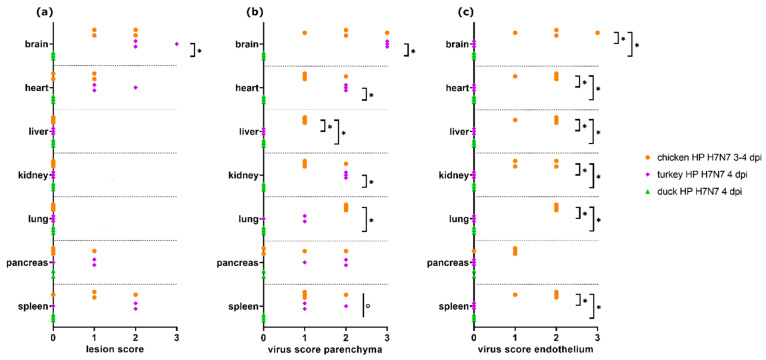
Lesion scores (**a**) and virus scores for parenchyma (**b**) and endothelium (**c**) in chickens, turkeys, and ducks (*n* = 3 or 4 each) infected with H7N7 highly pathogenic avian influenza virus (HPAIV) (Experiments 8 to 11). No necrotic lesions or antigens were traceable in the respective organs in ducks. Significant differences (*p* ≤ 0.05) between two groups as detected with Kruskal–Wallis tests followed by Dunn’s post hoc tests are marked with asterisks (*), significant differences (*p* ≤ 0.05) between all three groups as detected by Kruskal–Wallis test but not significant with Dunn’s post-test between two groups are marked with circles (°).

Semiquantitative scores were compared in chickens, turkeys, and ducks infected with the same H7N7 HPAIV strain and necropsied at 3–4 dpi (Experiments 8 to 11, *n* = 3 or 4 each; Figure 3).

Significant differences were detected for necrotizing encephalitis (Kruskal–Wallis test, *p* = 0.0048) comparing the three species. Dunn’s post-test showed significantly higher lesion scores in turkeys as compared with ducks for the brain (*p* = 0.0216). Moreover, turkeys showed a tendency towards higher lesion scores in the heart, pancreas, and spleen than chickens and ducks.

For parenchyma virus scores, the three groups showed significant differences in the brain, heart, lung, liver, kidney, and spleen (Kruskal–Wallis test, each *p* < 0.05), but none in the pancreas. Dunn’s post-test revealed significantly higher virus scores in turkeys as compared with ducks for brain, heart, and kidney as well as chickens compared with ducks for the lung. For the liver, ducks and turkeys had significantly lower virus scores than chickens (each *p* < 0.05).

Unlike chickens, ducks and turkeys did not display endothelial viral antigen in any of the examined organs. Statistically significant differences between turkeys and chickens as well as between ducks and chickens were detected for the brain, heart, lung, liver, kidney, and spleen (Kruskal–Wallis test with Dunn’s post-test, each *p* < 0.05).

### 3.4. Scoring of H7N7- and H5N8B-Infected Chickens

**Figure 4:**
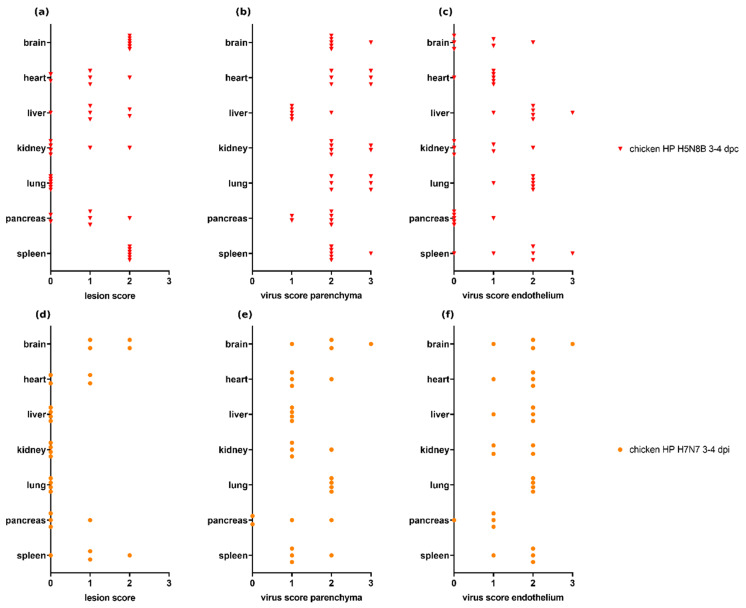
Lesion scores and virus scores for parenchyma and endothelium in chickens infected with H5N8B highly pathogenic avian influenza virus (HPAIV) (Experiment 6.2, *n* = 6) (**a**–**c**) and H7N7 HPAIV (Experiments 8 and 9, *n* = 4) (**d**–**f**). H5N8B-infected chickens are sentinel animals, so the time since virus exposition is given as days post-contact (dpc) for selected subgroups and as days post-inoculation (dpi) for H7N7-infected chickens.

Both sentinel chickens infected with H5N8B HPAIV and necropsied at 3–4 dpc (Experiment 6.2, *n* = 6) and chickens inoculated with H7N7 HPAIV and necropsied at 3–4 dpi (Experiments 8 and 9, *n* = 4) showed necrotizing lesions, lymphoid depletion, and viral antigen in many of the examined organs (Figure 4). Comparing both independent experiments, a trend towards more severe necrotizing hepatitis and lymphoid depletion and/or necrosis in the spleen (Figure 4a,d) and a more abundant distribution of parenchymal virus antigen in the heart, kidney, and spleen (Figure 4b,e) can be observed in chickens infected with H5N8B HPAIV than in H7N7-infected animals. Endothelial viral antigen was present in all selected organs with a variable and comparable distribution in both groups, except a trend towards higher scores in the heart in H7N7-infected chickens.

### 3.5. Scoring of H7N7- and H5N8B-Infected Ducks

**Figure 5:**
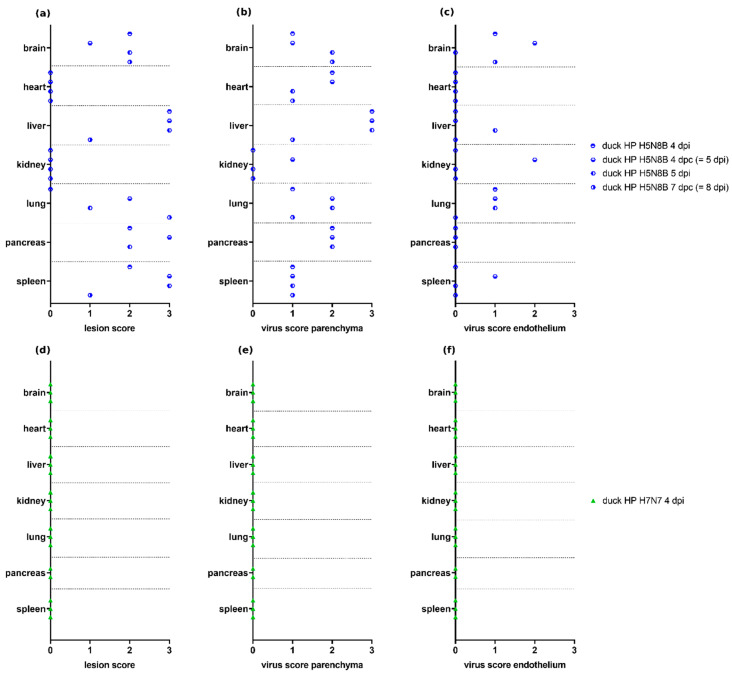
Lesion scores and virus scores for parenchyma and endothelium in ducks infected with H5N8B highly pathogenic avian influenza virus (HPAIV) (Experiment 6.1, group “duck HP H5N8B 4–8 dpi,” listed as individual animals) (**a**–**c**) and H7N7 HPAIV (Experiment 11, group “duck HP H7N7 4 dpi”) (**d**–**f**). No necrotic lesions or antigen were seen in the examined organs in H7N7-infected ducks. The H5N8B-infected duck with the highest lesion score in the lung suffered from additional aspergillosis, which may have contributed to lesion severity in this animal. The H7N7-infected ducks and two of the H5N8B-infected ducks were inoculated oculonasally, and time points are given as dpi for those animals, accordingly. The other two H5N8B-infected ducks are sentinel animals, and, therefore, time points are given as dpc for those ducks additionally (calculated as one day less than dpi).

Ducks infected with H5N8B HPAIV and necropsied at 4–8 dpi (Experiment 6.1, *n* = 4) showed lesion scores of variable severity in the brain, lung, pancreas, liver, and spleen, as well as a wide range of parenchymal and endothelial virus scores, whereas no lesions and viral antigen were observed in the selected organs of ducks infected with H7N7 HPAIV and necropsied at 4 dpi (Experiment 11, *n* = 3) (Figure 5).

### 3.6. Comparison of H5N8B-Infected Chickens and Ducks

**Figure 6:**
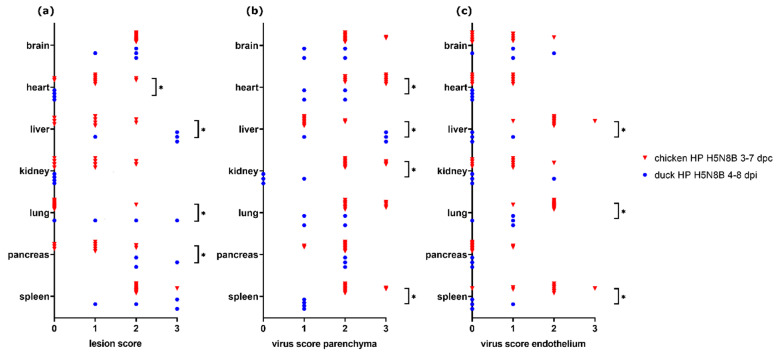
Lesion scores (**a**) and virus scores for parenchyma (**b**) and endothelium (**c**) in chickens (*n* = 10) and ducks (*n* = 4) infected with H5N8B highly pathogenic avian influenza virus (HPAIV) (Experiments 6.1 and 6.2). Significant differences (*p* ≤ 0.05) as detected by Mann–Whitney U test are marked with asterisks (*).

A comparison of lesion score and virus score for chickens and ducks infected with the same H5N8B HPAIV-strain (Experiments 6.1 and 6.2) and necropsied at 3–7 dpc (chickens, *n* = 10) and 4–8 dpi (ducks, *n* = 4) was made (Figure 6). Nearly all ducks showed severe necrotizing hepatitis, whereas, in most of the chickens, only mild or no necrotizing hepatocellular lesions were observed. Other significantly higher lesion scores for ducks were found in the pancreas, whereas lesion score in the heart was significantly higher in chickens (Mann–Whitney U test, each *p* < 0.05).

The severity of necrotizing pneumonia was significantly different between the groups (Mann–Whitney U test, *p* = 0.031). However, the duck with the highest lesion score suffered from additional aspergillosis, which may have contributed to lesion severity in this animal.

Parenchymal virus scores were significantly higher in ducks for the liver (Mann–Whitney U test, *p* = 0.0182), but in chickens for heart, kidney, and spleen (Mann–Whitney U test, each *p* < 0.05). Endothelial virus score was significantly higher in chickens for liver, lung, and spleen (Mann–Whitney U test, each *p* < 0.05) compared with ducks.

More details for the data which are shown in Figure 6 are given in Figure S2 for chickens and Figure S3 for ducks, where scores are arranged according to the different time points after infection (dpi/dpc).

### 3.7. Comparison of Different Time Points within H7N7-Infected Chickens

**Figure 7:**
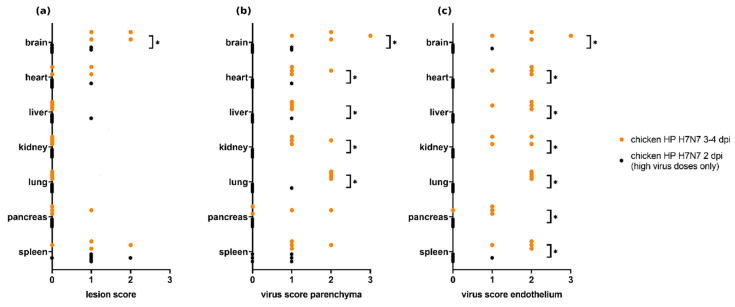
Lesion scores (**a**) and virus scores for parenchyma (**b**) and endothelium (**c**) in chickens infected with the same H7N7 highly pathogenic avian influenza virus (HPAIV) strain and necropsied at 2 dpi (Experiment 3, *n* = 6) and 3–4 dpi (Experiments 8 and 9, *n* = 4). Lower lesion and antigen scores are seen for chickens necropsied at 2 dpi. Significant differences (*p* ≤ 0.05) as detected by the Mann–Whitney U test are marked with asterisks (*).

Lesion scores and virus scores were compared for chickens infected with the same H7N7 HPAIV virus strain (clone or wild-type) and necropsied at 2 dpi (Experiment 3, *n* = 6) or 3–4 dpi (Experiments 8 and 9, *n* = 4), respectively (Figure 7). Significantly higher lesion scores were seen in chickens necropsied at 3–4 dpi in the brain (Mann–Whitney U test, *p* = 0.0333). Only low parenchymal virus scores and nearly no endothelial viral antigen were observed in chickens necropsied at 2 dpi compared with chickens necropsied at 3–4 dpi, with significant differences for parenchymal virus scores in the brain, heart, liver, kidney, and lung and for endothelial virus score in all compared organs (Mann–Whitney U test, each *p* < 0.05).

### 3.8. Comparison of Parenchyma Antigen Score and Virus Quantification

**Figure 8:**
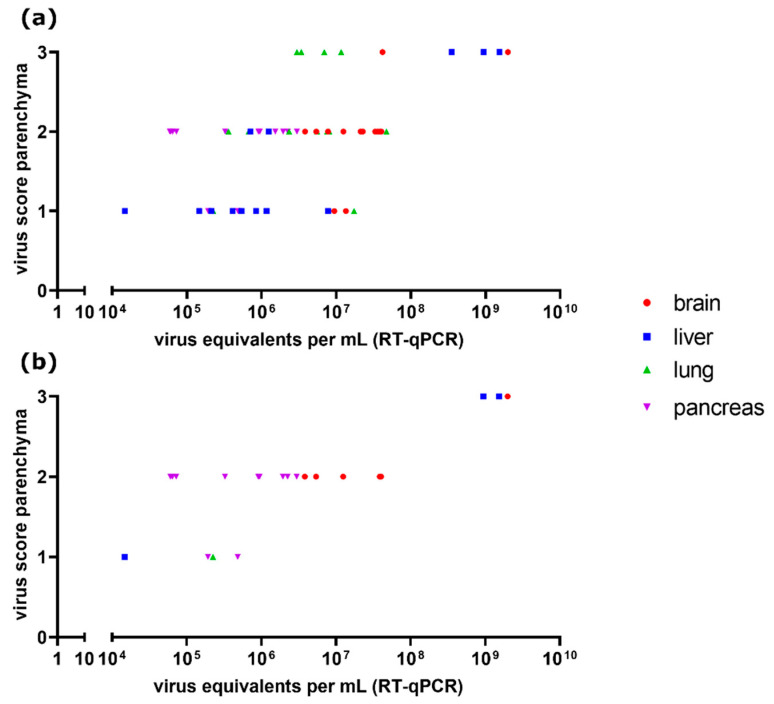
Parenchymal virus scores and viral RNA loads (in virus equivalents per mL as detected by reverse transcription-quantitative polymerase chain reaction (RT-qPCR)) for different organs of H5N8B highly pathogenic avian influenza virus (HPAIV) infected chickens (Experiment 6.2, *n* = 10, necropsied at 3–7 dpc) and ducks (Experiment 6.1, *n* = 4, necropsied at 4–8 dpi), including (**a**) and excluding (**b**) tissues with a display of endothelial viral antigen. A significant positive correlation (Spearman’s correlation analysis, *p* < 0.001 each) was detected for both cases. The correlation was higher for tissues with no endothelial viral antigen exclusively (*r* = 0.6938) than for tissues both with and without endothelial viral antigen (*r* = 0.4758).

For the H5N8B HPAIV-infected chickens (Experiment 6.2, necropsied at 3–7 dpc, group “chicken HP H5N8B 3–7 dpc,” *n* = 10) and ducks (Experiment 6.1, necropsied at 4–8 dpi, group “duck HP H5N8B 4–8 dpi,” *n* = 4), quantitative RT-qPCR data and antigen distribution scores based on immunohistochemistry were available (see Figure S3 in [42]). Correlation analysis of viral RNA loads and parenchymal antigen scores showed a significant positive correlation (Spearman’s correlation analysis, *p* < 0.001 each) for all tissues available regardless of additional endothelial immunoreactivity (*r* = 0.4758), and for tissues selected for the absence of endothelial viral antigen (*r* = 0.6938), the last-mentioned correlation being stronger than the first one (Figure 8).

## 4. Discussion

The first aim of this study was to develop an easily applicable, universal scoring system that allows a standardized histological evaluation of pathologic changes caused by different avian influenza viruses. Implementation of such a system allows to assess, compare, and statistically analyze results across different virus subtypes, hosts, tissues, and time points. As a second aim, such a comparative analysis was done in this study exemplarily.

A scoring system with a well-defined set of grades can help to achieve an increase in consistency and reproducibility [36,51]. This especially opens the way for the comparison of results between different experiments, scientists, and institutions.

By focusing on only seven tissues representing the organ systems hitherto known as being most commonly affected by AIV [10,11,12,13,14,16,18,19,20,21,24,41,42,44], the proposed pathotyping system allows effective and meaningful analysis of either large and small amounts of tissue samples. Nonetheless, it can be easily adapted for the analysis of additional organs, if required.

The proposed system is using a rather small array of four scoring categories. As shown previously, four to five categories are most commonly used and provide optimal results in terms of reproducibility and detection [28,29,36]. The condensation of the complex individual morphological diagnoses, which are normally generated by veterinary pathologists, into only four scores reflects the demand of virologists to focus on the most important lesions and statistically support or falsify hypotheses using mathematical models. However, users should be aware that scoring systems can be an oversimplification and erroneously hide findings with drastic negative effects on experiments such as unrelated co-infections. Therefore, scoring systems should be accompanied by information on other lesions such as unusual lesions and unrelated background lesions.

Furthermore, due to the great variability of AIV infections, seldom cases occur that cannot be clearly classified based on the more precise criteria stated above. In such cases, it is advised to rely on the more universal scoring criteria given or, in uncommon cases, even exclude outliers from scoring critically considering all data, rather than strictly following the suggested more detailed criteria.

Moreover, the consistency of results can be influenced by other factors such as the methods of tissue sampling and processing [51,52,53]. Therefore, to further optimize comparability, a standard procedure for sampling and processing is needed as well as a large-scale validation of the inter- and intra-observer repeatability and further score correlation to tissue pathobiology and/or other clinical aspects [36].

However, in most cases and with a sufficient sample size, the scoring system provides appropriate and well-defined criteria for examination and analysis of tissues, which can help to maintain consistency between different examiners.

Another objective of this study was to conduct a meta-analysis comparing different in-vivo influenza infection experiments using semiquantitative scores [41,42,44]. Generally, a variability in tissue tropism and pathology between different avian and non-avian species and viral subtypes is often observed [12,21,54].

For the data analyzed here, HP H7N7-infected turkeys showed slightly or significantly more severe necrotic lesions in the brain, heart, pancreas, and spleen compared with chickens and ducks (*p* = 0.0216 for brain score compared with ducks). Similar findings have been reported for another H7 subtype [55].

In selected tissues from chickens infected with HP H7N7 taken at 2 dpi, no or only mild lesions, low parenchyma virus scores and only sparse endothelial viral antigen were seen, whereas in tissues from chickens infected with a clone of the same HP H7N7 strain, but taken at 3–4 dpi, more severe lesions and higher virus scores were detected in a greater number of animals (*p* = 0.0333 for brain lesion score, *p* < 0.05 for five and seven organs for parenchymal and endothelial virus score, respectively). Similar findings were reported in other studies: H5N1-infected Pekin ducks showed an increase in lesion severity in various organs from 2–3 dpi up to 5 dpi, when the last of the animals were killed [56], and an increase of antigen distribution over many organs in the first days after infection was detected [18,56].

Altogether, differences in tissue tropism were seen for all three species: For all H7N7-infected chickens and turkeys shown here, lesions were predominantly seen in the brain, heart, pancreas, and spleen, whereas no, or in one chicken only, mild lesions were detected in the liver, kidney, and lung. Nonetheless, viral antigen was found in all compared organs except in ducks, suggesting systemic infection for chickens and turkeys. No endothelial viral antigen was seen in HP H7N7-infected turkeys and ducks, contrary to chickens. Endotheliotropism of HPAIV is very common in chickens and turkeys, but not in ducks [11,27,57], apart from a few exceptions [18,27,58], suggesting a pathogenesis for galliform species (including chickens and turkeys) different from that in ducks [27]. However, some virus strains seem to lack tropism for vascular endothelium in turkeys, similar to observations presented here and indicate another route of pathogenesis in those cases as well [59].

All chickens of the chosen experimental groups that were either infected with HP H5N8B or with HP H7N7 and necropsied at 3–4 dpi or dpc showed necrotizing lesions, lymphoid depletion as well as viral antigen in a wide range of organs. A tendency towards a higher severity of necrotizing hepatitis, more severe lymphoid necrosis, apoptosis, or depletion in the spleen and wider parenchymal virus antigen distribution in heart, kidney, and spleen could be observed in the chickens infected with HP H5N8B. This observation may be a possible hint towards increased virulence of HP H5N8B; however, due to the variability of infection routes in the re-analyzed experiments, this point needs further confirmation in prospective studies.

The examined HP H5N8B-infected Pekin ducks showed necrotic lesions and parenchymal antigen of varying degrees in many selected organs, whereas no lesions or parenchymal antigen were found in Muscovy ducks infected with HP H7N7. Therefore, the pathogenicity of HPAIV for ducks is highly dependent on virus strain and/or duck species. Although, in general, ducks often display less severe lesions, clinical symptoms and mortality compared with chickens [54,55,60,61,62], some strains show high pathogenicity for ducks and are able to cause necrotizing and inflammatory lesions as well as widespread antigen distribution. Such lesions are most commonly seen in association with some H5 virus strains [14,54,56,63,64,65]. Although pathological examination data for H7 in ducks is rather sparse, some H7 subtypes are reported to show higher pathogenicity for ducks and can be associated with clinical symptoms and mortality as well [66,67].

The endothelial viral antigen displayed in H5N8B-infected ducks but not in H7N7-infected ducks is a less frequent finding, as endotheliotropism of AIV is only occasionally reported in ducks contrary to chickens [18,27,58].

Compared to HP H5N8B-infected chickens, the Pekin ducks infected with the same virus strain showed especially severe necrotizing lesions in the liver and pancreas, whereas nearly all of the infected chickens, but none of the ducks had lesions in the heart (*p* < 0.05 each). Influenza virus antigen was found in the parenchyma of many organs in chickens and ducks, suggesting a systemic infection for both species. Even though those results should be interpreted keeping in mind the variability in time points and route of infection between the animals examined in this meta-analysis, the marked difference in lesion severity, especially in liver, pancreas, and heart, in animals necropsied at the time of death, no matter which day of the pathogenicity index experiments it occurred, suggests a difference in tissue tropism and pathogenesis for this H5N8B virus strain. Marked hepatic and pancreatic necrosis as characteristic lesions in Pekin ducks infected with the HP H5N8B virus strain were also seen in one of the other infection experiments used for the development of the proposed scoring system [43].

All comparisons made in this meta-analysis have to be evaluated cautiously since a confounding effect of the differences in the experimental design of the original studies may mimic true virus, strain, or host effects. Other studies have shown that infection dose [68], age at examination [14,69], and days after infection [18,56] can systematically influence the outcome of influenza virus infection. This point was addressed by manually selecting only the most comparable individuals from our previous studies wherever possible. Regarding the influence of the variation of the infection dose, only the range inducing comparable severe clinical symptoms and/or a high mortality rate was selected.

Regarding the correlation of virus loads detected by RT-qPCR or virus isolation and immunohistochemically detected antigen, some studies argue in favor of a certain correlation [56,70], whereas others reported no such correlation [18]. The results seen here for HP H5N8B-infected animals show a highly significant positive correlation between RT-qPCR results and immunohistochemical parenchyma virus score.

RT-qPCR analysis, unlike the immunohistochemical scoring system, semi-quantifies the overall amount of viral antigen in the tissue, irrespective of the exact localization, e.g., in endothelial or parenchymal cells. As it can be hypothesized that this may have a blurring effect on the relationship between RT-qPCR results and parenchyma antigen score, the correlation analysis was conducted twice, once including and once excluding animals with immunoreactive endothelia. A strengthening of correlation when excluding organs with additional positive endothelial virus score could be observed. However, the interpretation of this tendency is restricted by reduced sample size and variability of scores for the selected dataset and thus in need of further investigation, as well as the examination of organ-specific variations in correlation, which was not conducted due to similar limitations.

As discussed above, the results of this meta-analysis should be interpreted cautiously, mainly due to the small sample sizes and the variability in the study design of available experiments. Future studies should use a larger sample size for more robust results.

While further research will be necessary for its refinement, the proposed scoring system can be used as a valuable and convenient instrument to conduct a standardized analysis of AIV-induced histopathological and immunohistochemical changes. This tool enables comparison of results across a wide range of host species, viral strains and experimental conditions and helps to improve the significance of systematic histopathologic and immunohistochemical examination in future avian influenza research.

## Figures and Tables

**Table 1:** Lesion Score: Criteria for histopathologic scoring of avian influenza virus (AIV)-induced lesions.

Organ	Score 0	Score 1	Score 2	Score 3
all	no	mild	moderate	severe
	parenchymal necrotic/necrotizing inflammatory lesions			
brain	no	mild	moderate	severe
	necrotizing polioencephalitis/meningoencephalitis with/without gliosis and with/without perivascular lymphohistiocytic infiltration			
heart	no	mild	moderate	severe
	necrotizing myocarditis/myocardial necrosis with/without lymphohistiocytic infiltration			
lung	no	mild	moderate	severe
	(fibrino)necrotizing (broncho-)pneumonia/necrosis of parenchymal cells			
liver	no	mild	moderate	severe
	necrotizing hepatitis/hepatocellular necrosis with/without lobular/perivascular pattern and with/without lymphohistiocytic infiltration			
kidney	no	mild	moderate	severe
	necrosis of tubular epithelium with/without lymphohistiocytic infiltration			
pancreas	no	mild	moderate	severe
	necrotizing pancreatitis/pancreatic necrosis			
spleen	no	mild	moderate	severe
	necrosis/apoptosis of lymphoid and/or reticuloendothelial cells and/or lymphoid depletion			

**Table 2:** Definition of parenchymal cells used for the virus score.

Organ	Parenchymal Cells
all	functional cells of organ (e.g., epithelia)
brain	neurons, glial cells
heart	cardiomyocytes
lung	bronchiolar and parabronchiolar epithelia, pneumocytes, alveolar macrophages *
liver	hepatocytes
kidney	tubular epithelium
pancreas	exocrine pancreatocytes
spleen	reticular cells, round cells (lymphocytes, dendritic cells, macrophages)

* Since they cannot be distinguished from detached pneumocytes type II in hematoxylin and eosin-stained slides.

**Table 3:** Virus score: Criteria for immunohistologic scoring of distribution of avian influenza virus (AIV)-antigen in parenchymal cells and parenchymal necrotic areas.

Organ	Score 0	Score 1	Score 2	Score 3
all	no	focal to oligofocal	multifocal	coalescing to diffuse
	antigen in parenchymal cells/necrotic parenchymal areas			
further criteria for orientation and classification of inconclusive cases				
brain	-	less than 5% immunoreactive area and up to 0.75 clusters/low-power field *	less than 5% immunoreactive area and more than 0.75 and up to 5 clusters/low-power field * or 5%–20% immunoreactive area and up to 5 clusters/low-power field *	more than 20% immunoreactive area and/or more than 5 clusters/low-power field *
heart	-	less than 10% immunoreactive area and less than 2 immunoreactive cells/ high-power field *	less than 10% immunoreactive area and 2–10 immunoreactive cells/ high-power field *	at least 10% immunoreactive area and/or more than 10 immunoreactive cells/ high-power field *
lung	-	less than 5% immunoreactive area and less than 3 immunoreactive cells/ high-power field *	less than 5% immunoreactive area and at least 3 immunoreactive cells/ high-power field *	at least 5% immunoreactive area
liver				
- necrotic areas immunoreactive	-	less than 5% immunoreactive area	5%–14% immunoreactive area	at least 15% immunoreactive area
- mainly single cells immunoreactive	-	less than 3 immunoreactive cells/ high-power field *	3–10 immunoreactive cells/ high-power field *	more than 10 immunoreactive cells/ high-power field *
kidney	-	less than 2 clusters/ low-power field * and up to 2% immunoreactive area	at least 2 clusters/ low-power field * and up to 5% immunoreactive area with single clusters	5% immunoreactive area with coalescing clusters or more than 5% immunoreactive area
pancreas	-	less than 5% immunoreactive area and up to 1 cluster/ low-power field *	less than 5% immunoreactive area and more than 1 cluster/ low-power field * or 5%–24% immunoreactive area	at least 25% immunoreactive area
spleen				
- necrotic areas immunoreactive	-	less than 2% immunoreactive area	2%–14% immunoreactive area	at least 15% immunoreactive area
- mainly single cells immunoreactive	-	up to 15 immunoreactive cells/ high-power field *	16–40 immunoreactive cells/ high-power field *	more than 40 immunoreactive cells/ high-power field *

* average out of four representatively chosen fields; low-power field: ×100 magnification (field number = 18, field of view area = 2.54 mm^2^); high-power field: ×400 magnification (field number = 18, field of view area = 0.159 mm^2^).

## Data Availability

The data presented in this study are available in this article, in associated articles cited in “2.1 AIV Infection Experiments” or on request from the corresponding author.

## Supplementary Materials

The following are available online at https://www.mdpi.com/article/10.3390/v13050868/s1, Scheme S1: Flow diagram of scoring system development, Table S1: Overview of infection experiments used for the development of the proposed semiquantitative scoring system, Figure S1: Scoring of lesion severity and antigen distribution in different organs across high pathogenic avian influenza virus (HPAIV) infected animals, Figure S2: Lesion scores and virus scores for parenchyma and endothelium in sentinel chickens infected with H5N8B HPAIV arranged by different days post-contact (dpc) with inoculated animals, Figure S3: Lesion scores and virus scores for parenchyma and endothelium in ducks infected with H5N8B HPAIV arranged by different days post-inoculation (dpi). References [39,40,41,42,43,44,45,46] were cited in the supplementary materials.
