# Supplementary material for: A Semiquantitative Scoring System for Histopathological and Immunohistochemical Assessment of Lesions and Tissue Tropism in Avian Influenza
Source: Viruses. 2021 May 9;13(5):868. doi: 10.3390/v13050868 (PMC8151536; doi:10.3390/v13050868)
Supplement: Supplementary file 1 [file viruses-13-00868-s001.zip › viruses-1179359-supplementary.pdf]

viruses

MDPI

Supplementary Materials: A Semiquantitative Scoring System for Histopathological and Immunohistochemical Assessment of Lesions and Tissue Tropism in Avian Influenza

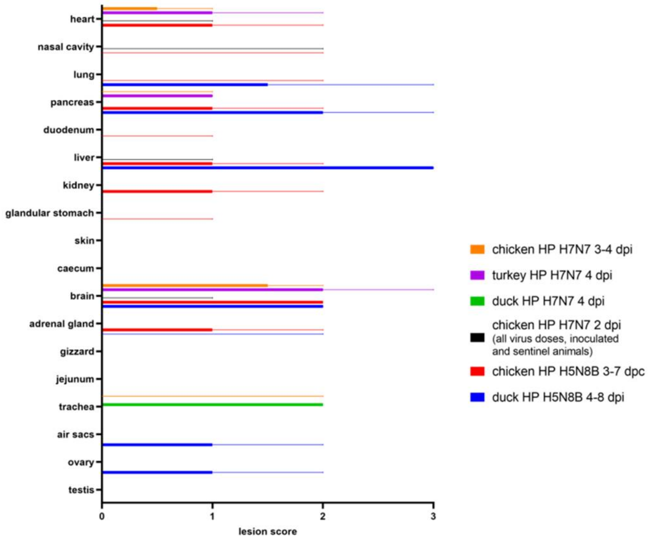

(a)

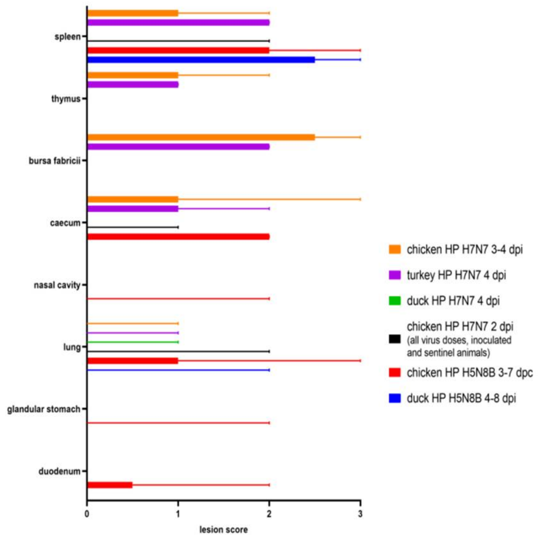

(b)

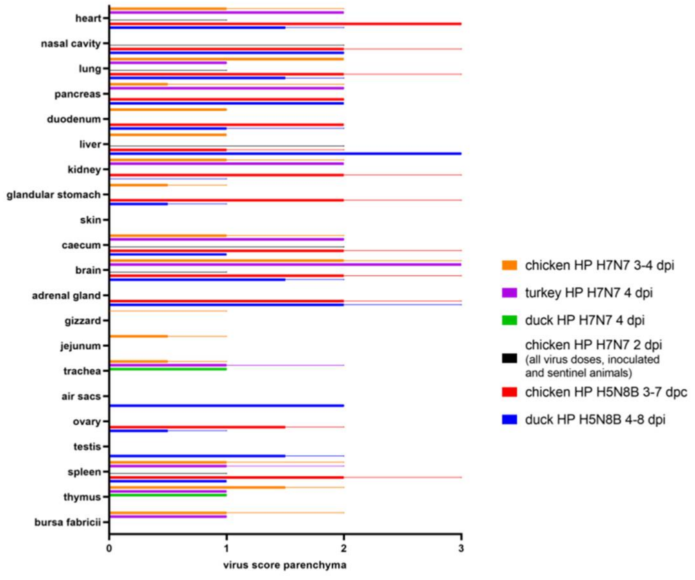

(c)

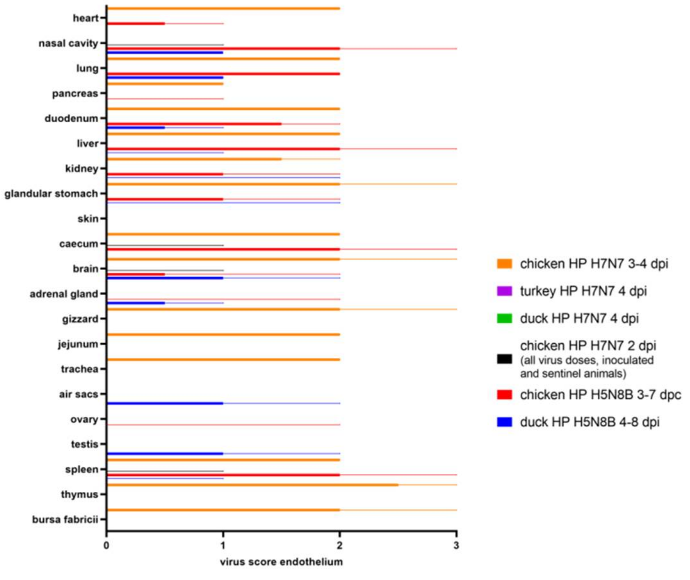

(d)

Table S1. Scoring of lesion severity and antigen distribution in different organs across highly pathogenic avian influenza virus (HPAIV) infected animals: (**a**) necrosis/necrotizing inflammation (lesion score), (**b**) lymphatic necrosis, apoptosis and/or lymphoid depletion (lesion score), (**c**) antigen in parenchymal cells (virus score parenchyma) and (**d**) antigen in endothelial cells (virus score endothelium). Median with range.(**a**), (**b**): Lesions were rated as follows: 0 = no lesion, 1 = mild lesion, 2 = moderate lesion, 3 = severe lesion; (**c**): Antigen distribution was rated as follows: 0 = no antigen, 1 = focal antigen, 2 = multifocal antigen, 3 = coalescing to diffuse antigen; (**d**): Antigen distribution was rated as follows: 0 = no antigen, 1 = single blood vessels immunoreactive, 2 = multiple blood vessels immunoreactive, 3 = diffuse immunoreactivity

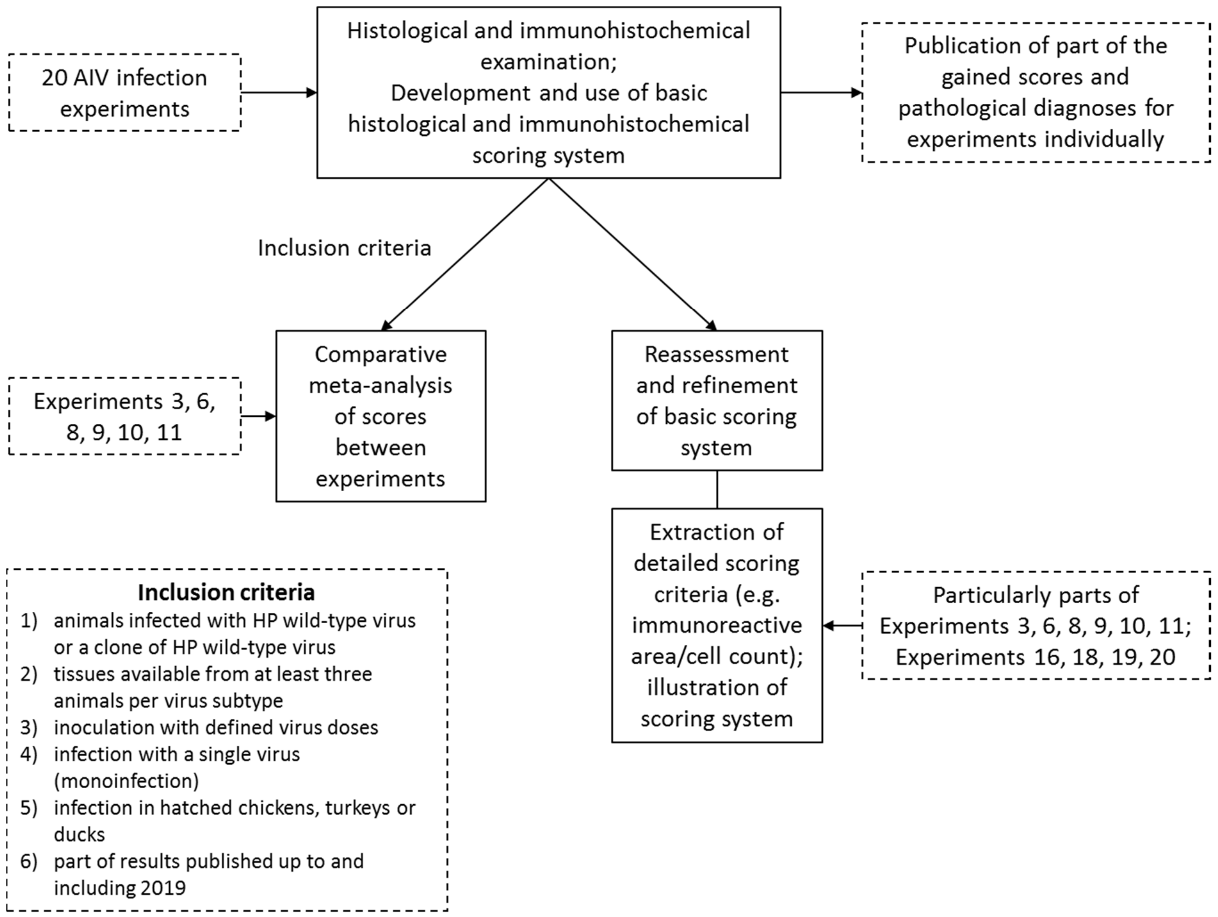

Scheme S1. Flow diagram of scoring system development. Tissue samples of 20 experimental avian influenza virus (AIV) infection experiments (details see Table S1) were examined histologically and immunohistochemically and a basic scoring system was developed and used in this process. Part of the gained results from the histological and immunohistochemical examination was published for some of the studies individually. In this study, data from selected experiments was compared in a meta-analysis. Furthermore, the basic scoring system was critically reassessed and refined taking all listed experiments into account. In this process a part of data and tissue slides was used for specification of detailed scoring criteria and illustration of histological and immunohistochemical scores in particular. HP: high pathogenic.

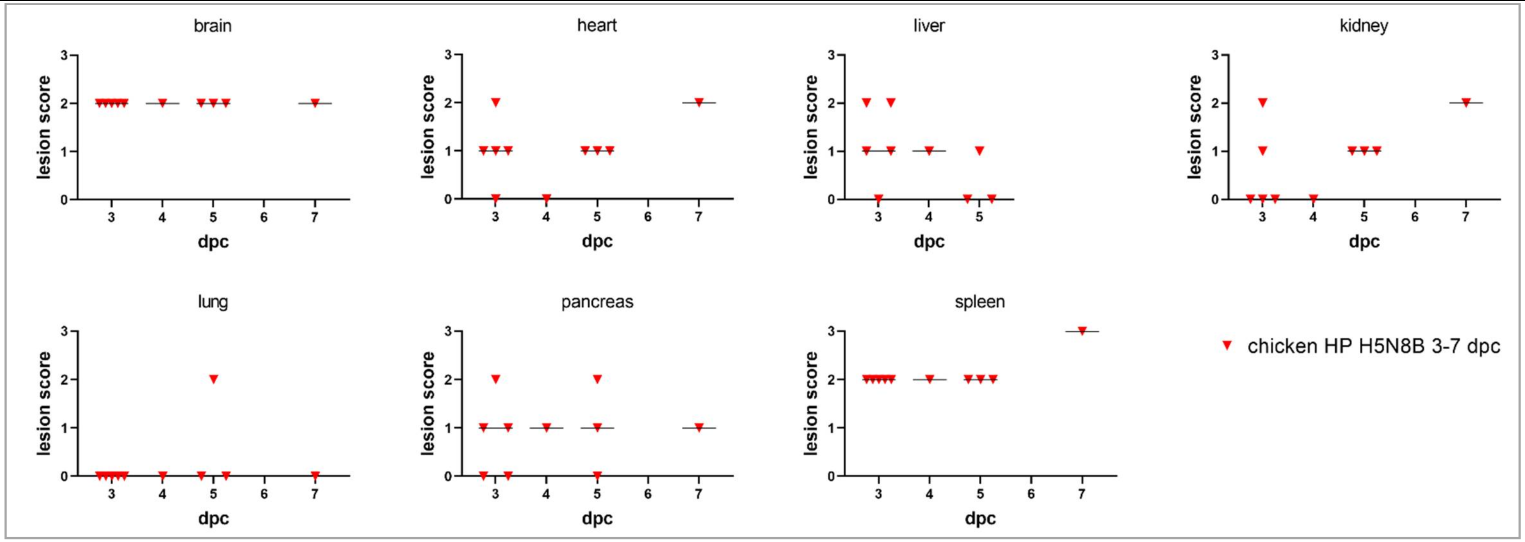
brain
heart
liver
kidney
lung
pancreas
spleen
lesion score
dpc
0
1
2
3
4
5
6
7
chicken HP H5N8B 3-7 dpc

(**a**)

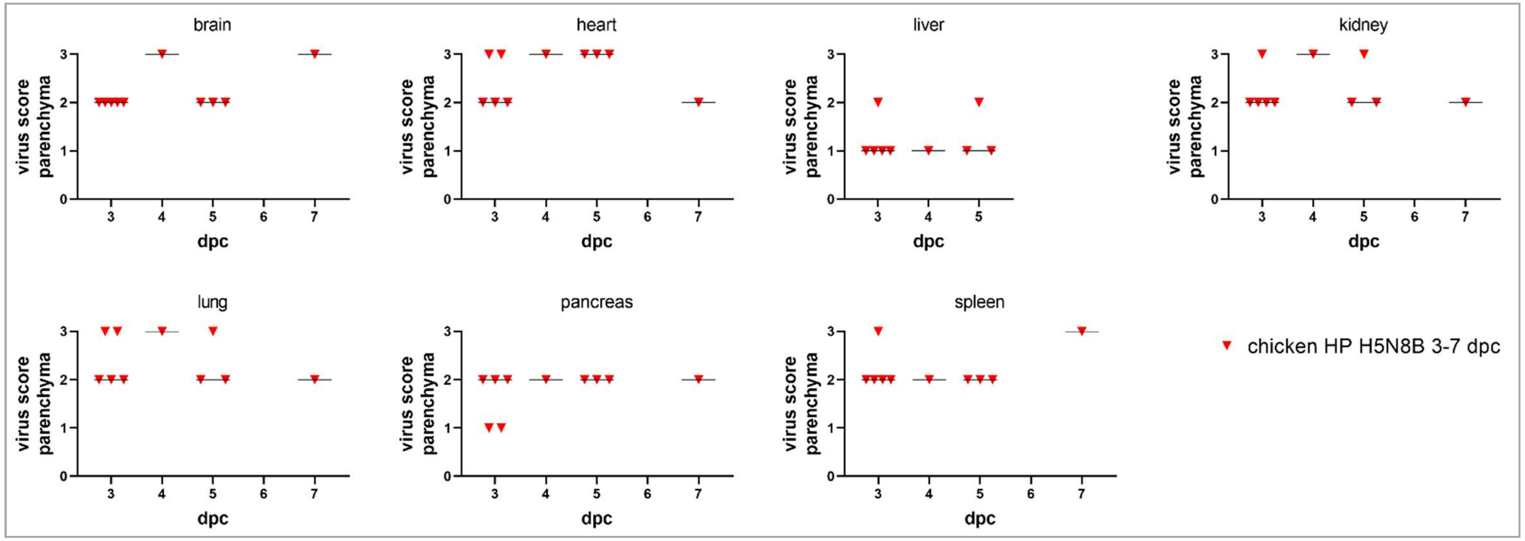
brain
heart
liver
kidney
lung
pancreas
spleen
virus score parenchyma
dpc
0
1
2
3
4
5
6
7
chicken HP H5N8B 3-7 dpc

(**b**)

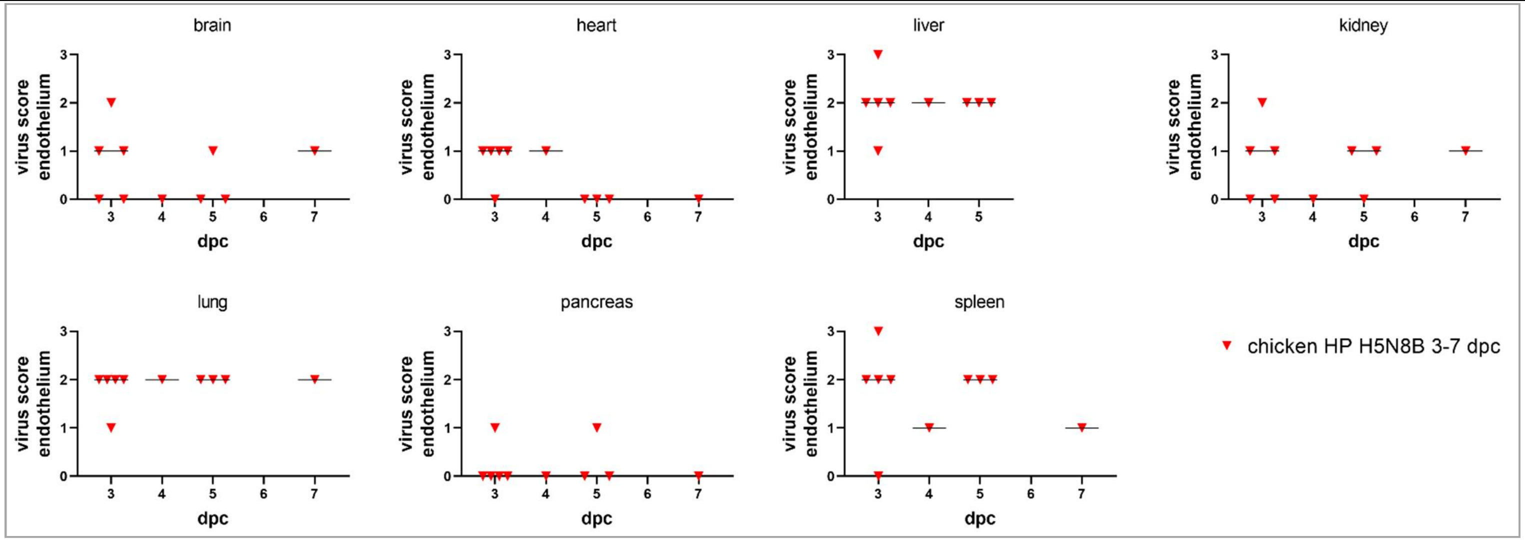

(**c**)

Figure S2. Lesion scores (**a**) and virus scores for parenchyma (**b**) and endothelium (**c**) in sentinel chickens infected with H5N8B highly pathogenic avian influenza virus (HPAIV) (Experiment 6.2, *n* = 10, group "chicken HP H5N8B 3–7 dpc", also see Figure 6) arranged by different days post contact (dpc) with inoculated animals. Scatter plot with median (line).

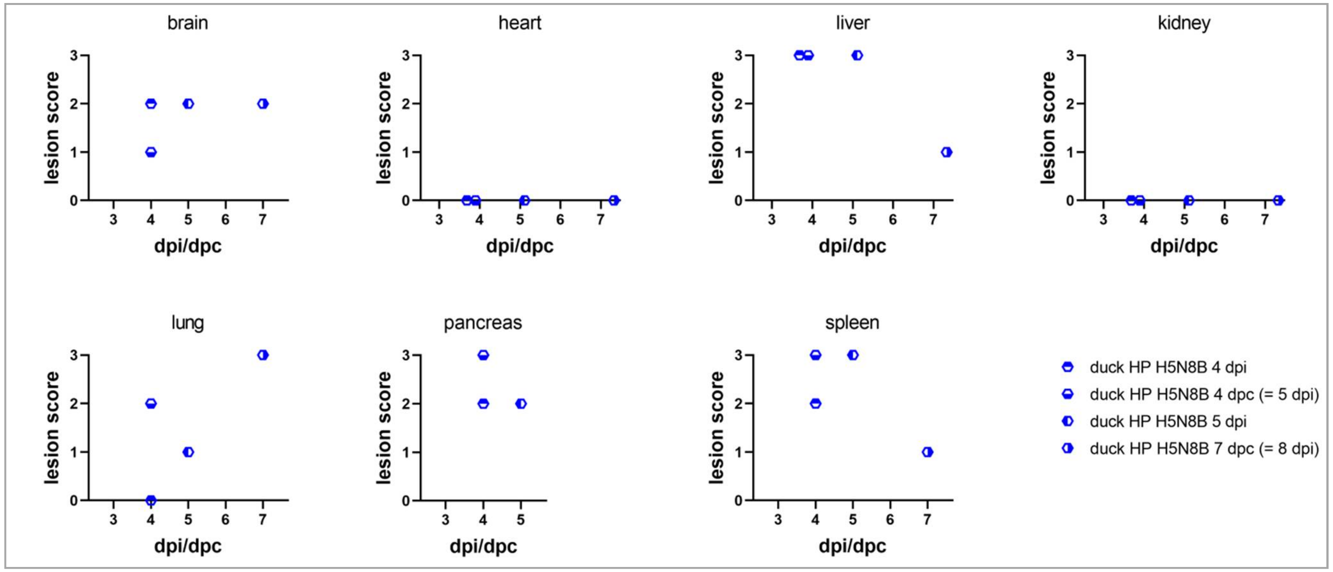
brain
heart
liver
kidney
lung
pancreas
spleen
lesion score
dpi/dpc
duck HP H5N8B 4 dpi
duck HP H5N8B 4 dpc (= 5 dpi)
duck HP H5N8B 5 dpi
duck HP H5N8B 7 dpc (= 8 dpi)

(**a**)

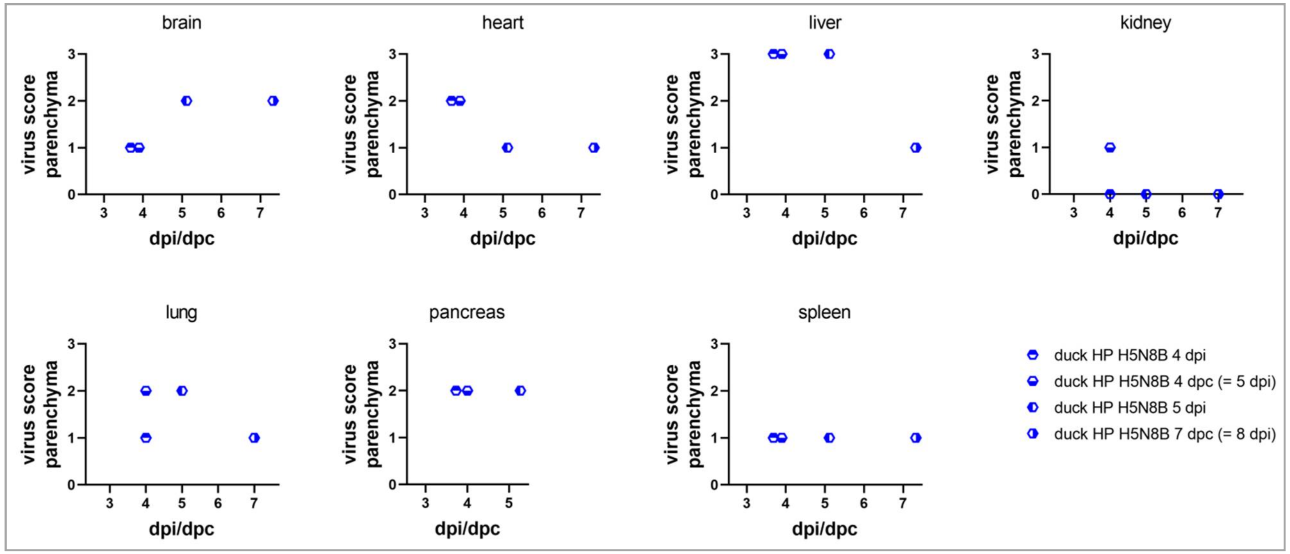
brain
heart
liver
kidney
lung
pancreas
spleen
virus score parenchyma
dpi/dpc
duck HP H5N8B 4 dpi
duck HP H5N8B 4 dpc (= 5 dpi)
duck HP H5N8B 5 dpi
duck HP H5N8B 7 dpc (= 8 dpi)

(**b**)

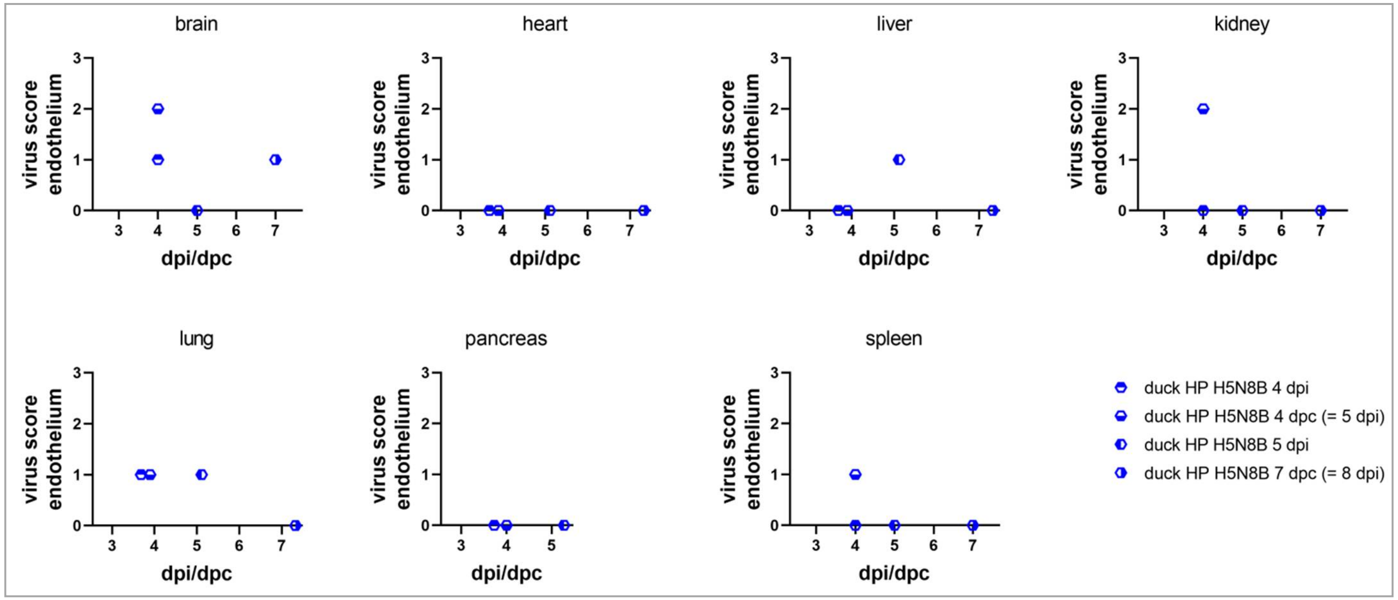

(**c**)

Figure S3. Lesion scores (a) and virus scores for parenchyma (b) and endothelium (c) in ducks infected with H5N8B highly pathogenic avian influenza virus (HPAIV) (Experiment 6.1, n = 4, group "duck HP H5N8B 4–8 dpi", also see Figure 5 and Figure 6) arranged by different days after infection. Two of the ducks were inoculated oculonasally and time points are given as days post inoculation (dpi) for those animals. The two other ducks were associated at 1 dpi as sentinel animals. Therefore, time points after infection are plotted as days post contact (dpc) for the sentinel ducks, and dpc can be calculated as one day less than dpi (e.g., 4 dpc at 5 dpi). Scatter plot.

Table S1. Overview of infection experiments used for development of proposed semiquantitative scoring system.

**No.**	**1**	**2**	**3**	**4**	**5.1**	**5.2**
virus subtype	H9N2, H5N1	H5N1, H9N2	H7N7	H7N7	H5N8A	H5N8A
wt/modif	wt HP + modif	wt HP + modif	wt HP, wt LP	wt HP, wt LP	wt HP	wt HP
virus strain[1]	A/chicken/Egypt/AR755/2013 (H9N2 LP) A/chicken/Egypt/AR236/2015 (H5N1 HP)	A/chicken/Egypt/AR755 /2013 (H9N2 LP) A/chicken/Egypt/AR236 /2015 (H5N1 HP)	A/chicken/Germany/ AR1385/2015 (HP) A/chicken/Germany/ AR915/2015 (LP)	A/chicken/Germany/AR1385/ 2015 (HP) A/chicken/Germany/AR915/2 015 (LP)	A/turkey/Germany-MV/ R2472/2014	A/turkey/Germany-MV/ R2472/2014
species[2]	chicken	ferret	chicken	ECE	duck	goose
animal number (n)[2]	11	3	48	24	4	4
age at infection	8 wk	n.r.	6 wk	10 + 14 days	6 to 7 mo	6 to 7 mo
infection dose	$10^{5.0}$ $TCID_{50}$	$10^{5.0}$ $TCID_{50}$	10^{1} to 10^{6} EID_{50} (HP), 10^{6} EID_{50} (LP) or both	10^{1} to $10^{5.7}$ EID_{50} (HP/LP), 10^{6} EID_{50} (LP)	10^{6} $TCID_{50}$	10^{6} $TCID_{50}$
infection route	conjunctival, intranasal	intranasal	oculo-oronasal, sen-tinel	allantoic	oculonasal	oculonasal
samples[3] taken at	2 dpi	up to 4 dpi	2 dpi	up to 4 dpi	2 and 4 dpi	2 and 4 dpi
registration number	LALLF MV 7221.3-1-033/15	LALLF MV 7221.3-1-033/15	LALLF MV 7221.3–1.1–039/17	LALLF MV 7221.3–1.1–039/17	LVL MV/TSD/7221.3-1.1-023/ 13 and/or 7221.3-2.2-002/09 (for exp. 5 to 7)	LVL MV/TSD/7221.3-1.1-023/ 13 and/or 7221.3-2.2-002/09 (for exp. 5 to 7)
antibody for IHC	anti-NP	anti-NP	anti-MP	anti-MP	anti-NP	anti-NP
used for me-ta-analysis	no	no	in parts (wt HP)	no	no	no
used for images	no	no	No	no	no	no
used for detailed specification of scoring criteria	no	no	in parts	no	no	no
part of results pub-lished	yes	yes	Yes	yes	yes	yes
first author	Naguib	Naguib	Graaf	Graaf	Grund	Grund
year of publication	2017	2017	2018	2018	2018	2018
reference	[45]	[45]	[41]	[41]	[42]	[42]
comment	immunohistochemical scoring only	immunohistochemical scoring only	-	-	5.1, 5.2 and 5.3 were housed together; mainly	5.1, 5.2 and 5.3 were housed together; mainly

					IHC scoring	IHC scoring

Table S1. continued: Overview of infection experiments used for development of proposed semiquantitative scoring system.

**No.**	**5.3**	**6.1**	**6.2**	**7**	**8**	**9**
virus subtype	H5N8A	H5N8B	H5N8B	H5N8B	H7N7	H7N7
wt/modif	wt HP	wt HP	wt HP	wt HP	clone wt HP, clone wt LP, clone wt LP modif	clone wt HP, clone wt LP modif
virus strain[1]	A/turkey/Germany-MV/R2472/2014	A/tufted_duck/Germany/AR8444-L01987/2016	A/tufted_duck/Germany/AR8444-L01987/2016	A/tufted_duck/Germany/AR8444-L01987/2016	A/chicken/Germany/AR1385/2015 (HP) A/chicken/Germany/AR915/2015 (LP)	A/chicken/Germany/AR1385/2015 (HP) A/chicken/Germany/AR915/2015 (LP)
species[2]	chicken	duck	chicken	mouse	chicken	chicken
animal number (n)[2]	3	4	10	5	8	16
age at infection	adult	6 to 7 mo	adult	4 wk	6 wk	6 wk
infection dose	-	10^6 $TCID_{50}$	-	10^2 $TCID_{50}$	10^5 PFU	10^5 PFU
infection route	sentinel	oculonasal, sentinel	sentinel	intranasal	oculonasal	oculonasal
samples[3] taken at	4 to 7 dpc	4 to 8 dpi	3 to 7 dpc	9 to 14 dpi	4 dpi	2 to 4 dpi
registration number	LVL MV/TSD/7221.3-1.1-023/13 and/or 7221.3-2.2-002/09 (for exp. 5 to 7)	LVL MV/TSD/7221.3-1.1-023/13 and/or 7221.3-2.2-002/09 (for exp. 5 to 7)	LVL MV/TSD/7221.3-1.1-023/13 and/or 7221.3-2.2-002/09 (for exp. 5 to 7)	LVL MV/TSD/7221.3-1.1-023/13 and/or 7221.3-2.2-002/09 (for exp. 5 to 7)	LALLF MV 7221.3-1-060/17	LALLF MV 7221.3-1-060/17
antibody for IHC	anti-NP	anti-NP	anti-NP	anti-NP	anti-NP	anti-NP
used for meta-analysis	no	yes	yes	no	in parts (wt HP)	in parts (wt HP)
used for images	no	yes	yes	no	no	in parts
used for detailed specification of scoring criteria	no	in parts	in parts	no	in parts	in parts
part of results published	yes	yes	yes	yes	yes	no

first author	Grund	Grund	Grund	Grund	Scheibner	-
year of publication	2018	2018	2018	2018	2019	-
reference	[42]	[42]	[42]	[42]	[44]	-
comment	5.1, 5.2 and 5.3 were housed together; mainly IHC scoring	6.1 and 6.2 were housed together	6.1 and 6.2 were housed together	+ NC group (n=3)	+ one NC group (n=4) for exp 8+9	+ one NC group (n=4) for exp 8+9

Table S1. continued: Overview of infection experiments used for development of proposed semiquantitative scoring system

No.	10	11	12	13	14	15
virus subtype	H7N7	H7N7	H9N2	H4N2, H5N1, H5N2, H7N7	H5N8B, different LP	H9N2, H9N8
wt/modif	clone wt HP, clone wt LP, modif	clone wt HP, clone wt LP, modif	wt LP + modif	modif	wt HP (+wt LPs/HP)	9 wt viruses
virus strain[1]	A/chicken/Germany/ AR1385/2015 (HP) A/chicken/Germany/ AR915/2015 (LP)	A/chicken/Germany/ AR1385/2015 (HP) A/chicken/Germany/AR915/2015 (LP)	A/turkey/Germany/AR1685/2016	A/quail/California/D113023808/2012 (H4N2) A/swan/Germany/R65/2006 (H5N1) A/chicken/Italy/8/1998 (H5N2) A/chicken/Germany/AR1385/2015 (H7N7)	A/tufted duck/Germany/ AR8444-L01987/2016 (H5N8B HP)	see [46]
species[2]	turkey	duck	turkey	chicken	duck	ECE
animal number (n)[2]	12	12	18	22	29	20
age at infection	6 wk	2 to 3 wk	6 wk	6 to 8 wk	14 mo, 18 mo, 5 wk	14 days
infection dose	10^5 PFU	10^5 PFU	$10^{5.7}$ PFU	10^5 PFU	10^6 EID_{50}	n.r.
infection route	oculonasal	oculonasal	oculonasal	oculonasal	nasal, sentinel	n.r.
samples[3] taken at	4 dpi	4 dpi	4 dpi	(2 to) 4 dpi	up to 34 dpi	4 dpi
registration number	LALLF MV 7221.3-1-060/17	LALLF MV 7221.3-1-060/17	LALLF M-V; 7221.3–1.1–051-12	LALLF M-V; 7221.3-1.1-051-12	LALLF MV 7221.3-1.1-025/18 and LALLF MV/TSD/7221.3-2-006/19	LALLF 7221.3-2-009/19
antibody for IHC	anti-NP	anti-NP	anti-MP	anti-NP	anti-MP	anti-MP
used for meta-analysis	in parts (wt HP)	in parts (wt HP)	no	no	no	no
used for images	no	no	no	no	no	no
used for detailed specification of scoring criteria	in parts	in parts	no	no	no	no
part of results published	yes	yes	yes	yes	yes	yes
first author	Scheibner	Scheibner	Blaurock	Gischke	Koethe	Parvin

year of publication	2019	2019	2020	2020	2020	2020
reference	[44]	[44]	[39]	[40]	[43]	[46]
comment	-	-	-	+ NC group (n=3)	partly natural pre-exposition with LP, re-infection with HP	+ mock-infected (n=2); immuno-histochemical scoring only

Table S1. continued: Overview of infection experiments used for development of proposed semiquantitative scoring system.

**No.**	**16**	**17**	**18**	**19**	**20**
virus subtype	H4N2	H7N7	H4N2, H5N1	H7N7	H5N8
wt/modif	modif	Clone wt LP modif	modif	modif	modif
virus strain[1]	-	-	-	-	-
species[2]	chicken	turkey	chicken	chicken	chicken
animal number (n)[2]	21	24	24	12	24
age at infection	6 wk	6 wk	6 wk	6 wk	6 wk
infection dose	10^5 PFU	10^5 PFU	10^5 PFU	10^5 PFU	10^5 PFU
infection route	oculonasal	oculonasal	oculonasal	oculonasal	oculonasal
samples[3] taken at	4 dpi	4 dpi	3 to 4 dpi	4 dpi	4 dpi
registration number	LALLF MV 7221.3-1-060/17	LALLF MV 7221.3-1-060/17	LALLF MV 7221.3-1-060/17	LALLF MV 7221.3-1-060/17	LALLF MV 7221.3-1-060/17
antibody for IHC	anti-MP	anti-NP	anti-MP	anti-MP	anti-MP
used for meta-analysis	no	no	no	no	no
used for images	in parts	no	in parts	no	no
used for detailed specification of scoring criteria	yes	no	yes	yes	yes
part of results published	no	no	no	no	no
first author	-	-	-	-	-
year of publication	-	-	-	-	-
reference	-	-	-	-	-
comment	-	-	-	-	-

Abbreviations: No.: experiment number in manuscript; HP: high pathogenic; LP: low pathogenic; wt: wild type; modif: reassortant, recombinant or genetically modified viruses; ECE: embryonated chicken egg; wk: weeks; mo: months; PFU: plaque forming units; EID_{50}: embryo infectious dose 50; $TCID_{50}$: tissue culture infective dose 50; dpi: days post inoculation, dpc: days post contact, NC: negative control; exp: experiment; n.r.: not reported; IHC: immunohistochemistry; MP: influenza A-matrixprotein; NP: influenza A-nucleoprotein. [1]only original wildtype viruses; [2]only animals subjected to histopathologic and/or immunohistochemical examination including scoring; [3]for histopathologic and immunohistochemical examination.
